# Spring Fever

**Published:** 1875-04

**Authors:** 


### "SPRING FEVER."

Upon the approach of warm weather, children—and grown folks, too, for that matter—become dull, with a feeling of lassitude akin to laziness. Most of us have experienced the feeling, and have spoken of it as "spring fever." This influance is likely produced by our continuing the full meals of strong, rich food to which we have subjected ourselves during the cold winter, and which was necessary to keep up the temperature of our bodies and so make us capable of resisting cold. But, upon the advent of the warm, pleasant weather of spring, we require a different character of food, and less of it, to meet the demands of the system. Hence it comes that, before such a change has been made, or has been considered necessary, we find ourselves distended with too much food, more than is required, so that we feel dull and sluggish, much as we imagine an anaconda must feel after having lunched upon a fair sized bullock.

Now, many people imagine this feeling of "spring fever" is attributable to "biliousness," and deem a large pill of blue mass and a brisk cathartic necessary to relieve the system from a superfluous quantity of bile. We have seen mothers call their children in from play upon a beautiful spring day, and administer a Thompsonian dose of lobelia, to each one of them, until the contents of their stomachs were deposited in the family wash-bowl, as an offering to gentle spring. Some mothers still think that it is quite as necessary to have a renovation of the stomach and bowels of the little folks, as it is to clean the house, and they count quite as much upon the annual family puke as they do upon the househould duty referred to.

If people would only refrain from the administration of the lobelia, blue pill, salts, and sulphur and molasses, to themselves and little ones, we are sure they will recover quite as quickly from the annual attack of "spring fever," and leave in the minds of their little children no dread of the approach of warm weather, knowing that their little stomachs must pay the penalty in receiving the nauseous drugs above enumerated.

